# Interaction between leukocyte aldo-keto reductase 1C3 activity, genotypes, biological, lifestyle and clinical features in a prostate cancer cohort from New Zealand

**DOI:** 10.1371/journal.pone.0217373

**Published:** 2019-05-24

**Authors:** Nishi Karunasinghe, Eva Symes, Amy Gamage, Alice Wang, Pam Murray, Shuotun Zhu, Megan Goudie, Jonathan Masters, Lynnette R. Ferguson

**Affiliations:** 1 Auckland Cancer Society Research Centre (ACSRC), Faculty of Medical and Health Sciences (FM&HS), The University of Auckland, Auckland, New Zealand; 2 Urology Department, Auckland City Hospital, Auckland, New Zealand; 3 Emeritus Professor, FM&HS, The University of Auckland, Auckland, New Zealand; National Health Research Institutes, TAIWAN

## Abstract

**Introduction:**

Aldo-keto reductase 1C3 (AKR1C3) is known for multiple functions including its catalytic activity towards producing extra-testicular androgen. The present study is towards understanding interaction between biological, lifestyle and genetic impacts of AKR1C3 and their influence on clinical factors in a prostate cancer (PC) cohort from New Zealand (NZ).

**Method:**

Characteristics of 516 PC patients were collected from the Auckland Regional Urology Facility, NZ. These men were genotyped for the *AKR1C3* rs12529 single nucleotide polymorphism (SNP). The leukocyte AKR1C3 activity was measured in a sub-cohort. Variability of leukocyte AKR1C3 activity between biological, lifestyle and clinical features as well as correlation between biological and clinical features were assessed with and without genetic stratification.

**Results:**

The leukocyte AKR1C3 activity was associated with age at diagnosis (0.51 vs 0.34 μM coumberol units for >69y vs ≤69y, P = 0.03); and with anatomic stage/prognostic grouping among the *AKR1C3* rs12529 CC genotype carriers (0.50 vs 28 μM coumberol units among low- and high-risk groups respectively, P = 0.02). Significant correlation between leukocyte AKR1C3 activity and age at PC diagnosis was also observed (correlation coefficient 0.20 and P = 0.02). Ever- smoking impacted both age and PSA at PC diagnosis among *AKR1C3* rs12529 GG and CG genotype carriers respectively. Age at diagnosis significantly correlated with PSA at diagnosis in the main (correlation coefficient 0.29, and P<0.001) and sub-cohorts (correlation coefficient 0.24, and P = 0.01); and those carrying the *AKR1C3* rs12529 CG and GG genotypes in both the main (correlation coefficient 0.30, and P<0.001 and correlation coefficient 0.35, and P<0.001 respectively) and sub-cohorts (correlation coefficient 0.43, and P<0.001 and correlation coefficient 0.39, and P = 0.06 respectively); but not with those carrying the CC genotype.

**Conclusions:**

Age dependent PSA thresholds in PC screening could have been valid only in men carrying the *AKR1C3* rs12529 CG and GG genotypes in this NZ cohort.

## Introduction

Prostate Cancer (PC) is the most common non-skin cancer among men in developed countries [1, 2]. In New Zealand (NZ) there were 3199 PC registrations and 607 PC-related deaths in 2012[3]. The status of PC risk varies between individuals in terms of the patient’s lifestyle and biological characteristics [4–6]. For PC management purposes, it is important to differentiate between men carrying indolent cancers from those with high-risk cancers. Routine assessments for PC include the digital rectal examination and the serum prostate-specific antigen (PSA) blood test, and a subsequent biopsy to confirm diagnoses. However, PC screening with PSA is currently debated due to its low specificity [7]. According to Merriel et al 2018 *e*valuations on performance of the PSA-based screening for diagnosis of both asymptomatic and symptomatic PC stands equivocal [8]. There are no specific serum PSA levels that are defined as normal/abnormal for men in any racial or ethnic group [2].

The serine protein PSA is produced subsequent to the androgen receptor (AR)-regulated expression of the PSA (*kallkirein-related peptidase 3* (*KLK3*)) gene, while action of androgens are mediated by the AR [9]. Meanwhile, *AKR1C3* is among the genes that show increased expression in advancing PC tissue [10]. The AKR1C3 enzyme is involved in reducing many aldehyde and ketone groups to their respective alcohols and is also known to catalyze the production of prostaglandins and extra-testicular androgens, including testosterone and dihydrotestosterone (DHT) [11]. AKR1C3 inhibitors have been shown to reduce PC cell growth in both *in vivo* and *in vitro* models, and making castrate resistant PC cell lines more sensitive to the AR antagonist enzalutamide treatment [12]. Recent research has shown a negative association with serum PSA levels in men carrying the G allele of the *AKR1C3* rs12529 C>G polymorphism in exon 1 of the *AKR1C3* gene which leads to a histidine to glutamine change in the AKR1C3 protein [4]. Meanwhile, allele C of this gene has been associated with increased PC-specific mortality among patients treated with androgen deprivation therapy (ADT) [13], while the same allele has been associated with increased cancer-specific symptoms if not treated with ADT [14].This same *AKR1C3* rs12529 C allele has been associated with an increased risk of high-risk PC [5]. Using AKR1C3 promoter constructs of the HepG2 cells, it has been shown that the promoter activity of this gene is associated with promoter the SNP rs3763676,with allele A leading to a 2.2 fold increased activity when treated with DHT in comparison to allele G [15]. The rs11551177 SNP A>G in exon 2 of the *AKR1C3* gene, leads to a glutamic acid to glycine change, and the G allele is associated with lower serum testosterone levels [16]. Increased testosterone and DHT dependant transactivation of AR has been recorded in *in vitro* cell cultures transfected with the *AKR1C3* gene [17]. AKR1C3 is also found to be supporting the stability of factors that regulate AR activity [18]. It is reported that the TMPRSS2-ERG fusion protein, which is well known for its expression in 70% of PCs [19], drives AKR1C3 expression by binding to the *AKR1C3* gene promoter region in PC cell cultures [10]. Our recent comparison of high-risk PC diagnosis pattern between US and NZ cohorts has shown a disparity between the two centres, especially among men with an ever-tobacco smoking lifestyle and carrying one or two G alleles on the *AKR1C3 rs12529* polymorphism [20]. We are of the view that delayed diagnosis of high-risk PC among NZ ever-smokers carrying the *AKR1C3 rs12529* G allele is due to its association with lower PSA levels compounded by lower PSA screening in NZ. Therefore, the PSA screening debate [7] could at least partially be due to variation of AKR1C3 activity among individuals, leading to difference in levels of extra-testicular androgen production.

AKR1C3 protein expression has been recorded in many human tissues including the leukocytes [21, 22]. The current study therefore aims to understand the interaction between AKR1C3 activity, *AKR1C3* rs12529 SNP genotypes, biological and lifestyle features, and clinical factors in a PC patient cohort from NZ.

## Material and methods

### Patient recruitment and data collection

The patient cohort considered here was from the ‘Genomic studies on Prostate Cancer’ study (ethics reference NTY/05/06/037), carried out at the University of Auckland in collaboration with the Urology Department, Auckland City hospital. The recruitment process involved inviting men of any ethnicity with positive biopsies for PC from the Auckland Regional Urology Registry (Auckland, Middlemore, and North Shore hospitals). Recruitment was carried out at the Green Lane Outpatient’s Clinic, in Green Lane, Auckland, the Manukau Super Clinic in Manurewa, and the North Shore hospital in Takapuna. Recruitment was restricted to men between 45–90 years of age attending the clinics for follow up before or after the surgery, hormonal or radiation therapy, chemotherapy, or those on active surveillance or watchful waiting. Patient recruitment took place between October 2006 and December 2013. Initially patients were recruited within one year of diagnosis, if they had not undergone any treatment for PC other than radical prostatectomy (RP). In 2008, the criterion was relaxed to include all patients with malignancies but within one year of diagnosis. In September 2010, the timeframe for recruitment was altogether removed. A total of 517 men were recruited from NZ to the study from which a sub-cohort of 155 recruited between September 2010 and December 2013 was considered for leukocyte AKR1C3 activity measurement. Patient recruitment from NZ was carried out with informed written consent under the Northern B (former Northern Y) ethics approval NTY/05/06/037. Clinical and pathology records of patients were evaluated at the hospital databases to collect age and PSA level at diagnosis, Gleason grade and disease stage [tumor-node-metastasis (TNM)].

Subsequently, patients were further stratified based on the disease stage/prognostic grouping followed the criteria defined by the 7^th^ edition of the American Joint Committee on Cancer (AJCC) abbreviated as I, IIA,IIB,III and IV as mentioned previously [20]. D’Amico et al retrospectively monitored a PC patient cohort that had undergone RP, and radiation implant with or without neoadjuvant ADT towards an outcome measure of PSA failure [23]. Based on these outcome measures, these authors were the first to stratify a high-risk PC patient category as those having a clinical tumour stage ≥T2C, or a PSA level of >20ng/ml, or a Gleason grade of ≥8 (equivalent to ≥ Stage IIB).

### SNP genotyping

At recruitment, patients provided a blood sample in an Ethylenediaminetetraacetic acid (EDTA) Vacutainer tube from Beckton Dickinson. DNA was extracted from 300μl of EDTA blood using a QIAamp genomic DNA kit (Qiagen, Hilden, Germany) following the manufacturers’ protocol with the aid of a fully automated QIAcube (Qiagen, Hilden, Germany). SNP genotyping was carried out using either the Sequenom MassArray system (Sequenom, San Diego, CA, USA) as described in Ferguson et al. 2010 [24] and subsequently the TaqMan SNP Genotyping Assay from Applied Biosystem (AB) using AB 7900 Real-Time PCR system [14, 24].

### Leukocyte AKR1C3 activity measurement

AKR1C3 activity measurement was based on a fluorometric coumberol assay adapted from Jamieson *et al* 2014 [21]. Coumberol (SN32425) used for the standard measures and the coumberone substrate (SN32424) and the AKR1C3 inhibitor (SN34037) were kindly provided by the in-house team at the Auckland Cancer Society Research Centre (ACSRC). All stock solutions for the coumberone substrate, the AKR1C3 inhibitor and coumberol were dissolved in DMSO, while coumberol standards were further diluted in PBS. Aliquots of an in-house produced AKR1C3 plasmid inserted LNCaP PC cell line (LNCaP+ cell line) was used as a positive AKR1C3 control at 1 million cells per ml concentration. This LNCaP+ cell line has been produced following protocols from Guise *et al* 2010 [25], to insert a plasmid encoding sequence-confirmed open reading frame for AKR1C3, which has been kindly provided by the AKR1C3 team. As the LNCaP prostate cancer cell line is known to have undetectable levels of AKR1C3 activity [26], 1 million cells per ml concentration of the LNCaP cell line was used as a negative control in each assay plate. These cells grown to confluence were stored in freeze media containing 75% phenol red free Minimum Essential Medium α from Gibco (Cat#41061–029), 15% sterile filtered fetal calf serum (from Moregate Australia & NZ) and 10% dimethyl sulfoxide (ECP Analytical reagent) in -80°C freezer until used.

A total of 155 blood samples collected with heparin anti-coagulant at recruitment were assayed for AKR1C3 activity. These samples were stored frozen at -80°C for up to 8 years. Blood samples were thawed at room temperature and immediately transferred onto an ice bath. These samples were mixed both by pipetting and vortexing with 4ml of cold phosphate buffered saline (PBS) pH 7.4, containing 2mM EDTA and 5% fetal calf serum to avoid clumping of the final cell pellet. This mixture was centrifuged at 1500 x g for 5min at 4°C. The supernatant was removed and the resultant cell pellet was washed twice more with PBS pH 7.4, containing 2mM EDTA and 5% fetal calf serum. Each cell pellet was mixed with 750μl of cold phenol red free Minimum Essential Medium α from Gibco (Cat#41061–029) and 180μl of sample was loaded in to four wells each on a black solid bottom Nunc F96 MicroWell Plate (Cat.# 137101). Positive and negative controls were also loaded to four wells each. To two of the wells with sample or positive/negative controls, the AKR1C3 inhibitor was added to provide a final concentration of 10μM in 200μl final volume. The plates were placed in a plate shaker (Eppendorf Thermomixer C) for 5sec at 300 rpm and were incubated at 37°C for 1 hr in the dark before the coumberone substrate was added to give a final concentration of 10μM in final 200μl volume in each well. The plates were placed in a plate shaker for 5sec at 300 rpm and incubated at 37°C overnight in the dark. 200μl aliquots of coumberol standards containing 0.5μM, 0.25μM, 0.125μM, 0.0625μM, 0.0312μM, 0.0156μM and blank PBS were made fresh prior to fluorescence measurements and were loaded in the standards wells in duplicate.

The fluorescence was measured at 390nm excitation and 510nm emission using the PelkinElmer Enspire Multimode Reader (PerkinElmer, Inc. USA) at room temperature, and the AKR1C3 activity was estimated as the amount of coumberol produced in wells with and without AKR1C3 inhibition. The difference in coumberol produced in wells with and without AKR1C3 inhibitor was considered as due to the total AKR1C3 activity produced by the leukocytes. Using a cell counting protocol, trypan blue stained cell suspension (10μl trypan blue: 10μl cell suspension) was assessed using Ventriplast 10 chamber counting slides, Cat.# 211710 to estimate the number of live cells per 1μl of cell suspension. Samples with less than 10,000 cell per 1ml were not considered in the final activity assessment. Therefore 14% of the initial samples assessed for AKR1C3 activity measurements were removed from analysis. Estimated coumberol produced per million leukocytes were considered equivalent to the AKR1C3 activity in tested samples. LNCaP cell line produced no detectable AKR1C3 activity in this assay. The median AKR1C3 activity in the LNCaP+ cell line was 0.13μM coumberol per million cells with 25^th^ and 75^th^ percentiles at 0.10 and 0.15. The coefficient of variation for inter-plate assay was 23.3% while that for the intra-plate assay was 13.8%

### Statistical analysis

In this analysis, those with a current or past tobacco smoking lifestyle were considered as ever-smokers, while the others were considered as never-smokers. Patients receiving luteinizing hormone agonists or anti-androgens were classified under ADT regardless of whether the treatment was short- (< six months) or long-term (≥six months). Brachytherapy on its own or with other radiation treatment were considered under radiation therapy (RT). Any type of prostatectomy (radical or robot assisted) was considered under RP. Active surveillance and watchful waiting categories were categorised per standard nomenclature.

Continuous variables were compared using the Kruskal-Wallis One Way Analysis of Variance on Ranks test as most data types were not normally distributed. Measurements for non-normally distributed data were provided as medians and 25% and 75% points. The Spearman Rank Order Correlation was used to analyse the non-linear correlation between continuous data sets. Categorical variables were tested with the Chi Square test. Trend lines for age and PSA at diagnosis was derived from the Polynomial, Cubic function f = y0+a*x+b*x^2+c*x^3. All statistical analysis were performed using SigmaPlot version 14.0 (Systat Software Inc.).

## Results

### Patient characteristics

General characteristics and clinical details of the main PC cohort and the cohort selected for the AKR1C3 activity assessment are given in Table 1.The main and sub-cohorts consisted of >96% Caucasians. Ethnicity data, BMI (at study entry), tobacco smoking lifestyle and alcohol consumption frequencies between the main and sub-cohorts are comparable. However, the sub-cohort was significantly older than the main cohort (median age 69y vs 66.6y, P<0.001). The clinical characteristics showed that the sub-cohort had a higher median PSA at prostate cancer diagnosis as compared to the main cohort (10.6ng/ml vs 8.6ng/ml, P = 0.02); and with a higher frequency of those with Gleason sum ≥8 compared to the main cohort (31.6% vs 20.9%, P = 0.02). TNM staging data was not available for 35.8% and 22.6% of the main and sub-cohorts respectively. According to the available data, the sub-cohort had a lower frequency of patients with ≥T2C (30.8% compared to the main cohort with 37.8%, P<0.001). However, the anatomic stage/prognostic grouping was comparable between the sub- and main cohorts with high-risk ≥IIB category being 45.9% and 49.1% of the respective cohorts (P = 0.48). Due to missing data with a TNM classification, further analyses with data stratified based on TNM was not tested. PC management options were also significantly different between the main and sub-cohorts (P<0.001). The sub-cohort recorded ADT as the main therapy (54.9%), while the main cohort had RP as the most common management option (54.6%). The time lag between recruitment and diagnosis was similar between the sub- and the main cohort (median 1y and 25th and 75th percentile being at 0 and 2y respectively for both groups, P = 0.73).

**Table 1:** Characteristics of patients from the main and sub-cohort.

Demographic and lifestyle		Main cohort	Sub-cohort	P value
Ethnicity number(percentage)	Any Caucasian*	495 (95.9)	130 (97.7)	0.55
	Asia/Pacific only	17 (3.3)	2 (1.5)	
	Not known	4 (0.8)	1 (0.7)	
Age in years at diagnosis (median, 25th and 75th percentile) [number]		66.6 (60.4,71.6) [n = 512]	69 (63, 75) [n = 130]	<0.001
BMI at recruitment (median, 25th and 75th percentile)		27 (25, 30) [n = 512]	27 (25, 30) [n = 131]	0.99
Tobacco smoking (number and percentage)	Ever smoker	287 (55.6)	87 (65.4)	0.11
	Never smoker	226 (43.80)	45 (33.8)	
	Not known	3 (0.6)	1 (0.8)	
Alcohol consumption	Yes	366 (70.9)	89 (67.0)	0.42
	No	149 (28.9)	43 (32.3)	
	Not known	1 (0.2)	1 (0.7)	
Time lag between diagnosis and recruitment (median, 25th and 75th percentile) [number]		1 (0, 2) [509]	1(0, 2)[130]	0.73
**Clinical**				
PSA at diagnosis ng/ml (median, 25^th^ and 75^th^ percentile)		8.6 (5.8,15.0) [n = 468]	10.6 (6.1, 19) [129]	0.02
Gleason sum (number and %)	<8	407(78.9)	90 (67.7)	0.02
	≥8	108(20.9)	42 (31.6)	
	Not known	1(0.2)	1 (0.7)	
TNM staging (n = 331)	≤T2C	139 (27.0)	62 (46.6)	<0.001
	≥T2C	192 (37.2)	41 (30.8)	
	Not known	185 (35.8)	30 (22.6)	
Anatomic stage/prognostic group number and %) [23, 27]	Low-risk <Stage IIB	254 (49.1)	61 (45.9)	0.48
	High-risk ≥Stage IIB	262 (50.7)	71 (53.4)	
	Not known	1 (0.2)	1 (0.7)	
Management method (number and %)	Prostatectomy with or without other treatments	281 (54.6)	13 (9.8)	<0.001
	Neo- or adjuvant ADT with or without other treatments	153 (29.7)	73 (54.9)	
	RT with or without other treatments	123 (23.9)	55 (41.3)	
	WW	32 (6.2)	3 (2.3)	
	Active surveillance	21 (4.1)	11 (8.3)	

*indicates those with any Caucasian ancestry.

The main cohort and the sub-cohort recorded 9 (1.8%) and 1 (0.8%) patients with castrate- resistant PC.

### Genetic data

Genotype data recorded for the *AKR1C3* rs12529 SNP genotype (Table 2) for 380 men in the main cohort showed that genotype and allele frequency data were within the Hardy-Weinberg equilibrium.

**Table 2:** Genotype and allele frequencies of the AKR1C3 rs12529 SNP recorded for the study cohort.

Genotype numbers (% frequencies)			% allele frequency	
CC	CG	GG	C	G
121(31.8)	166 (43.7)	93 (24.5)	0.537	0.463

### AKR1C3 activity variation in the sub-cohort

The median leukocyte AKR1C3 activity level was 0.41 (25^th^ and 75^th^ percentiles at 0.21 and 0.73 respectively). When data were stratified between the *AKR1C3* rs12529 SNP genotypes, no statistically significant difference was seen between genotypes, except for the heterozygous genotype recording 24% higher median activity compared to the CC genotype and 19.8% higher median activity compared to the GG genotype (Table 3). The median PSA level at prostate cancer diagnosis in this sub-cohort also showed no statistically significant difference between genotypes (Table 3).

**Table 3:** AKR1C3 activity and PSA variation between the *AKR1C3* rs12529 genotypes in the sub-cohort.

	AKR1C3 activity				PSA ng/ml			
AKR1C3 rs12529 genotype	N	Median	25%	75%	N	Median	25%	75%
Sub-cohort								
CC	47	0.37	0.17	0.84	45	10.5	5.65	19.75
CG	57	0.48	0.30	0.80	59	10.5	6.30	16.65
GG	26	0.40	0.16	0.60	24	12.45	6.25	22.3
P value		0.15				0.94		

N = subject number

AKR1C3 activity data were further stratified between median age at diagnosis ≤69 and >69y, BMI>25 and ≤25, never- and ever-smokers, alcohol consumers and non-alcohol consumers (Table 4). Those with age at diagnosis at ≥69y showed a significantly higher leukocyte AKR1C3 activity compared to those at <69y (0.51 [25th and 75th percentiles at 0.25 and 0.78] vs 0.34 [25th and 75th percentiles at 0.17 and 0.65], P = 0.03), however, this variation was not significant after stratifying between the *AKR1C3* rs12529 genotypes. However, none of the other features (BMI, alcohol consumption, and tobacco smoking behavior) showed any significant variation in AKRIC3 activity both with and without the *AKR1C3* rs12529 genotype stratification (Table 4).

**Table 4:** AKR1C3 activity variation between biological and behavioral factors with and without stratification for the *AKR1C3* rs12529 genotype in the sub-cohort.

AKR1C3 rs12529 genotype group	N	Lifestyle/ ADT	Median	25%	75%	P value within each genotype	P value within All or genotype groups
All	59	Median Age≤69y	0.34	0.17	0.65		**0.03**
	71	Median Age>69y	0.51	0.25	0.78		
CC	21	Median Age≤69y	0.29	0.15	0.68	0.21	0.11
	25	Median Age>69y	0.43	0.21	0.90		
CG	25	Median Age≤69y	0.41	0.26	1.04	0.63	
	31	Median Age>69y	0.52	0.31	0.78		
GG	11	Median Age≤69y	0.25	0.11	0.42	0.13	
	12	Median Age>69y	0.52	0.17	0.70		
All	45	BMI>25	0.45	0.21	0.80		0.86
	84	BMI≤25	0.39	0.21	0.73		
CC	18	BMI>25	0.35	0.19	0.83	0.98	0.21
	28	BMI≤25	0.38	0.16	0.82		
CG	18	BMI>25	0.59	0.32	1.21	0.22	
	39	BMI≤25	0.39	0.25	0.73		
GG	9	BMI>25	0.21	0.08	0.58	0.14	
	17	BMI≤25	0.48	0.21	0.67		
All	43	Alcohol No	0.39	0.26	0.65		0.96
	88	Alcohol Yes	0.4	0.193	0.758		
CC	18	Alcohol No	0.37	0.25	0.55	0.90	0.48
	28	Alcohol Yes	0.36	0.16	0.93		
CG	16	Alcohol No	0.48	0.295	0.982	0.80	
	41	Alcohol Yes	0.48	0.28	0.795		
GG	6	Alcohol No	0.405	0.145	0.52	0.74	
	19	Alcohol Yes	0.31	0.17	0.68		
All	45	Never- smoker	0.45	0.23	0.81		0.37
	88	Ever -smoker	0.39	0.21	0.72		
CC	17	Never- smoker	0.35	0.17	0.96	0.97	0.31
	29	Ever -smoker	0.37	0.20	0.77		
CG	14	Never- smoker	0.49	0.34	1.14	0.34	
	43	Ever -smoker	0.48	0.26	0.76		
GG	11	Never- smoker	0.51	0.25	0.60	0.34	
	14	Ever -smoker	0.23	0.16	0.55		

N = subject number

This sub-cohort was further stratified between PSA level at diagnosis ≤20 or >20, Gleason sum <8 and ≥8, PC anatomic stage/prognostic group <IIB and ≥IIB, those receiving no ADT vs those receiving ADT, and those receiving RP or no RP, both with and without further stratification, based on the *AKR1C3* rs12529 genotype (Table 5). AKR1C3 activity was marginally higher among those carrying the *AKR1C3* rs12520 GG genotype and having PSA at diagnosis >20ng/ml compared to those with ≤20ng/ml group (0.55 [25th and 75th percentiles at 0.28 and 0.75] vs 0.25 [25th and 75th percentiles at 0.16 and 0.51], P = 0.06). Those with the *AKR1C3* rs12529 CC genotype recorded a lower median AKR1C3 activity among those with high-risk PC (≥Stage IIB) group compared to those with low-risk (stage <IIB) group (0.28 [25th and 75th percentiles at 0.14 and 0.49] vs 0.50 [25th and 75th percentiles at 0.27 and 1.03], P = 0.02). None of the other features showed any significant variation in AKRIC3 activity.

**Table 5:** AKR1C3 activity variation with clinical factors with and without stratification for the *AKR1C3* rs12529 genotype in the sub-cohort.

Group- All or AKR1C3 rs12529 genotype	N		Median	25%	75%	P value within each genotype	P value within All or genotype groups
All	35	PSA≤20	0.38	0.17	0.87		0.86
	96	PSA>20	0.40	0.21	0.72		
CC	36	PSA≤20	0.38	0.18	0.87	0.34	0.13
	10	PSA>20	0.35	0.10	0.67		
CG	46	PSA≤20	0.44	0.28	0.74	0.29	
	11	PSA>20	0.64	0.39	1.07		
GG	16	PSA≤20	0.25	0.16	0.51	0.06	
	9	PSA>20	0.55	0.28	0.75		
All	89	GS≤8	0.39	0.22	0.76		0.58
	39	GS>8	0.42	0.20	0.73		
CC	32	GS≤8	0.38	0.16	0.84	0.51	0.39
	14	GS>8	0.35	0.19	0.71		
CG	45	GS≤8	0.48	0.26	1.01	0.98	
	12	GS>8	0.50	0.33	0.75		
GG	12	GS≤8	0.28	0.18	0.52	0.49	
	13	GS>8	0.42	0.14	0.74		
All	60	Stage <IIB	0.47	0.27	0.84		0.10
	68	Stage ≥IIB	0.39	0.17	0.71		
CC	24	Stage <IIB	0.50	0.27	1.03	**0.02**	0.06
	22	Stage ≥IIB	0.28	0.14	0.49		
CG	30	Stage <IIB	0.46	0.32	0.82	0.84	
	27	Stage ≥IIB	0.48	0.23	0.81		
GG	6	Stage <IIB	0.26	0.15	0.54	0.45	
	19	Stage ≥IIB	0.42	0.16	0.68		
All	51	No ADT	0.38	0.19	0.72		0.78
	69	ADT	0.43	0.21	0.76		
CC	18	No ADT	0.38	0.16	0.78	0.84	0.55
	27	ADT	0.35	0.20	0.82		
CG	26	No ADT	0.39	0.30	0.72	0.50	
	25	ADT	0.52	0.26	0.97		
GG	7	No ADT	0.31	0.17	0.55	0.75	
	17	ADT	0.42	0.19	0.64		
All	116	No RP	0.415	0.223	0.737		0.63
	13	RP	0.34	0.175	0.85		
CC	42	No RP	0.37	0.167	0.827	0.85	0.24
	4	RP	0.37	0.193	1.065		
CG	51	No RP	0.48	0.31	0.81	0.46	
	6	RP	0.43	0.188	0.685		
GG	23	No RP	0.42	0.2	0.6	0.15	
	2	RP	0.14	0.11	0.17		

N = subject number

### Clinical characteristics of the main cohort

Median age at diagnosis was marginally higher among ever-smokers compared to never-smokers (68y [25th and 75th percentiles at 62 and 72y] vs 65y [25th and 75th percentiles at 59 and 71y, P = 0.05)] (Table 6). When stratified by genotypes, median age at PC diagnosis were 68.0y [25th and 75th percentiles at 61, 73], 67.5y [25th and 75th percentiles at 62, 72] and 66.0y [25th and 75th percentiles at 62.5, 71] respectively among men with the AKR1C3 rs12529 CC, CG and GG genotypes (P = 0.76) (Table 6). Age at diagnosis was not different between those with different smoking status among the *AKR1C3* rs12529 CC and CG genotypes either. However, for those with the GG genotype, a significantly higher age at diagnosis was recorded in ever- smokers compared to never-smokers (68 [25th and 75th percentiles at 64, 72] vs 64.5 [25th and 75th percentiles at 57.8, 68] y, P = 0.01). PSA levels at PC diagnosis recorded for the main cohort showed no significant difference between the *AKR1C3* rs12529 genotypes (Table 6) (CC = 9.6 [25th and 75th percentiles at 6.0, 15.6], CG = 8.6 [25th and 75th percentiles at 5.9, 14.6], GG = 9.3 [25th and 75th percentiles at 6.0, 15.9], P = 0.22). There was no significant difference in median PSA level between never- (7.5 [25th and 75th percentiles at 5.5, 13.6]) and ever- smokers (9.1 [25th and 75th percentiles at 6.3, 17.0], P = 0.22) in the main cohort. When data were stratified by both smoking status and genotype, those with the CG genotype recorded a significantly higher median PSA level at diagnosis among ever-smokers (8.9 [25th and 75th percentiles at 6.2, 16.7] compared to never-smokers (7.2 [25th and 75th percentiles at 5.8, 12.2]), P = 0.03). Although the AKR1C3 rs12529 GG genotype carriers recorded a higher PSA level at diagnosis among ever-smokers (11.4 [25th and 75th percentiles at 7.5, 20.0]) compared to never-smokers 6.4 [25th and 75th percentiles at 5.2, 11.6], this was statistically non-significant (P = 0.50).

**Table 6:** Variation of age and PSA at diagnosis with and without stratification for the *AKR1C3* rs12529 genotype and tobacco smoking lifestyle in the main cohort.

	Median age at diagnosis in years ±SD [25th and 75th percentiles] (subject number)				Median PSA at diagnosis [25th and 75th percentiles] (subject number)			
	Main	Smoking status		P value by smoking status	Main	Smoking status		P value by smoking status
		never-smoke	ever-smoke			never-smoker	ever-smoker	
All	66.0 [60, 71] (n = 512)	65.0 [59, 71] (n = 223)	68.0 [62, 72] (n = 287)	0.05	8.6 [5.9, 9.4] (n = 466)	7.5 [5.5, 13.6] (n = 394)	9.1 [6.3, 17.0] (n = 72)	0.22
CC	68.0 [61,73] (n = 120)	67.5 [58.3, 72] (n = 46)	68.5 [62.3, 73.8] (n = 74)	0.19	9.6 [6.0, 15.6] (n = 120)	9.8 [5.6, 16.0] (n = 46)	9.4 [6.4, 17.7] (n = 74)	0.38
CG	67.5 [62,72] (n = 166)	67.0 [61.8, 72] (n = 56)	68.0 [62, 73] (n = 109)	0.94	8.6 [5.9, 14.6] (n = 166)	7.2 [5.8, 12.2] (n = 114)	8.9 [6.2,16.7] (n = 52)	**0.03**
GG	66.0 [62.5,71] (n = 91)	64.5 [57.8, 68] (n = 34)	68 [64, 72] (n = 57)	**0.01**	9.3 [6.0, 15.9] (n = 91)	6.4 [5.2, 11.6] (n = 34)	11.4 [7.5, 20.0] (n = 57)	0.50
P value between genotypes	0.76	0.16	0.81		0.22	0.26	0.32	

### Correlation between clinical and non-clinical factors with and without genetic stratification of the sub- and main cohorts

**Fig 1:**
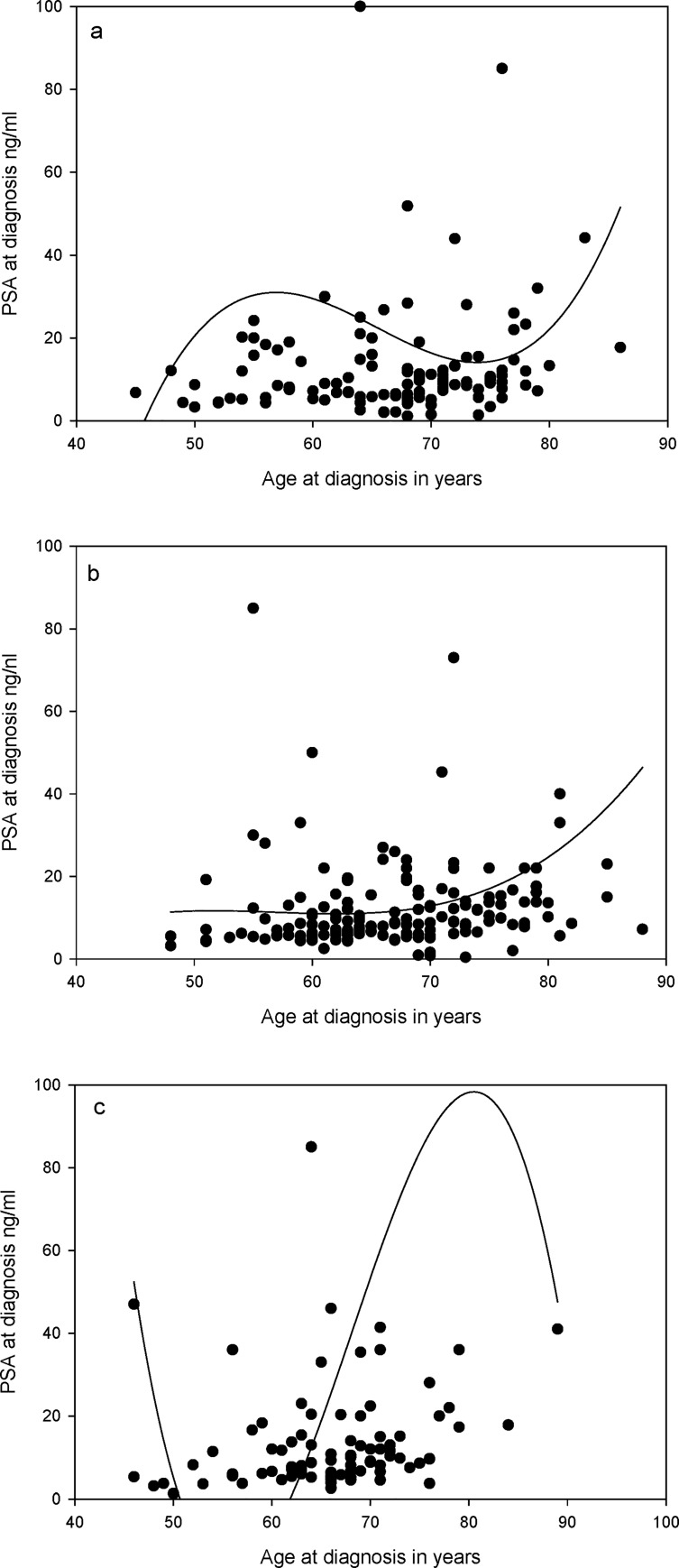
Trend lines between age and PSA at diagnosis for the *AKR1C3* rs12529 genotypes in the main cohort. (a—CC genotype, b—CG genotype and c—GG genotype).

Increasing age was negatively correlated with BMI in the sub-cohort (correlation coefficient -0.30 and P<0.01) as well as when stratified by the *AKR1C3* rs12529 CC (correlation coefficient -0.32, P = 0.04) and CG (correlation coefficient -0.33 and P = 0.01) genotypes (Table 7). In the main cohort, a significant correlation between age at diagnosis and BMI was not seen (Table 8). Age at diagnosis of the sub-cohort showed a correlation with AKR1C3 activity (correlation coefficient 0.20 and P = 0.02), PSA level at PC diagnosis (correlation coefficient 0.24 and P = 0.01) and Gleason sum (correlation coefficient 0.22 and P = 0.01) (Table 7). No correlation between the AKR1C3 activity and age at PC diagnosis was observed among stratified genotypes except for the *AKR1C3* rs12529 GG genotype showing a non-significant marginal correlation (correlation coefficient = 0.35 and P = 0.09). The *AKR1C3* rs12529 CG genotype group of the sub-cohort showed a significant correlation between age and PSA at diagnosis (correlation coefficient 0.43 and P<0.01) (Table 7). The AKR1C3 rs12529 GG genotype group of the sub-cohort showed a correlation trend between age and PSA at diagnosis (correlation coefficient 0.39 and P = 0.06). A significant correlation between the age and PSA at PC diagnosis was reproduced in the main cohort (correlation coefficient = 0.29, P<0.01) and the CG (correlation coefficient = 0.30, P<0.01) and GG genotypes (correlation coefficient = 0.35, P<0.01) (Table 8). As in the sub-cohort age and PSA at diagnosis did not correlate in the *AKR1C3* rs12529 CC genotype group. Trend lines for the variation between age and PSA at diagnosis stratified by the *AKR1C3* rs12529 genotype groups in the main cohort are shown in Fig 1A–1C. Those with the *AKR1C3* rs12529 CC genotype, a concentration of correlation points towards lower PSA levels around ages 65-75y was noted with the trend line. For the *AKR1C3* rs12529 CG and GG genotypes, a trend of increase is seen from 60y up to around 80y. Age at diagnosis also showed a correlation with the Gleason sum in the *AKR1C3* rs12529 CG genotype (correlation coefficient = 0.46 and P<0.01) in the sub-cohort. This was not reproduced in the main cohort, although in the main cohort, age at diagnosis and Gleason sum showed a marginal correlation within the GG genotype group (correlation coefficient 0.21 and P = 0.05) (Table 8). BMI and AKR1C3 activity showed no correlation with either PSA at diagnosis or the Gleason sum in the sub-cohort (Table 7). PSA at diagnosis showed a correlation with the Gleason sum in the sub-cohort (correlation coefficient 0.46 and P<0.01) as well as in the main cohort (correlation coefficient 0.27 and P<0.01) (Tables 7 and 8). Such significant correlations between PSA at diagnosis and Gleason sum were seen among those with the *AKR1C3* rs12529 CC and CG genotypes (correlation coefficient 0.48 and P<0.01 and correlation coefficient 0.45 and P<0.01 respectively) in the sub- cohort (Table 7). The correlation between PSA at diagnosis and Gleason sum was reproduced in the main cohort (correlation coefficient = 0.27 and P<0.01) and also remained when stratified by CC and CG genotypes (correlation coefficient 0.23 and P = 0.01 and correlation coefficient 0.29 and P<0.01 respectively) in the main cohort (Table 8). In addition in the main cohort the *AKR1C3* rs12529 GG genotype group also showed a correlation between PSA at diagnosis and Gleason sum (correlation coefficient 0.25 and P = 0.02).

**Table 7:** Spearman correlation statistics between BMI, AKR1C3 activity and clinical factors with and without the *AKR1C3* rs12529 genetic stratification in the sub-cohort. Statistics are given in the order of correlation coefficient, P value and the number assessed under each comparison.

	AKR1C3 rs12529 genotype	BMI	AKR1C3 activity	PSA at diagnosis	Gleason sum
Age at diagnosis	CC	-0.32	0.21	-0.01	0.02
		**0.04**	0.18	0.95	0.92
		43	44	44	44
	CG	-0.33	0.13	0.43	0.46
		**0.01**	0.33	**0.00**	**0.00**
		57	57	57	57
	GG	-0.25	0.35	0.39	0.14
		0.23	0.09	0.06	0.50
		24	24	24	24
	All	-0.30	0.20	0.24	0.22
		**0.00**	**0.02**	**0.01**	**0.01**
		124	125	125	125
BMI	CC		0.01	0.17	-0.16
			0.94	0.27	0.30
			43	43	43
	CG		-0.08	-0.02	-0.03
			0.55	0.86	0.84
			57	57	57
	GG		0.24	0.05	0.06
			0.25	0.83	0.77
			24	24	24
	All		0.02	0.05	-0.09
			0.83	0.56	0.35
			124	124	124
AKR1C3 activity	CC			-0.08	-0.18
				0.62	0.24
				44	44
	CG			0.05	0.02
				0.71	0.88
				57	57
	GG			0.17	0.13
				0.43	0.55
				24	24
	All			0.03	-0.08
				0.76	0.35
				125	125
PSA at diagnosis	CC				0.48
					**0.00**
					44
	CG				0.45
					**0.00**
					57
	GG				0.34
					0.10
					24
	All				0.46
					**0.00**
					125

**Table 8:** Spearman correlation statistics between BMI and clinical factors with and without the *AKR1C3* rs12529 genetic stratification in the main cohort. Statistics are given in the order of correlation coefficient, P value and the number assessed under each comparison.

	AKR1C3 rs12529 genotype	BMI	PSA	Gleason sum
Age at diagnosis	CC	-0.16	0.13	0.02
		0.09	0.16	0.85
		119	120	120
	CG	-0.12	0.30	0.12
		0.13	**0.00**	0.13
		165	165	165
	GG	0.02	0.35	0.21
		0.84	**0.00**	0.05
		91	91	91
	All	-0.06	0.29	0.12
		0.15	**0.00**	**0.01**
		509	466	511
BMI	CC		0.14	-0.10
			0.13	0.27
			119	119
	CG		-0.06	-0.07
			0.45	0.37
			166	166
	GG		-0.08	0.05
			0.48	0.63
			91	92
	All		0.04	-0.02
			0.45	0.62
			465	511
PSA at diagnosis	CC			0.23
				**0.01**
				121
	CG			0.29
				**0.00**
				166
	GG			0.25
				**0.02**
				91
	All			0.27
				**0.00**
				467

## Discussion

This study presents an analysis of interaction between AKR1C3 activity, *AKR1C3* rs12529 genotypes, biological and clinical features in a PC cohort from Auckland, NZ. The leukocyte AKR1C3 activity was measured only in a sub-cohort. Due to the sub-cohort showing significantly higher age and PSA at PC diagnosis as well as a higher PC severity as shown by Gleason sum data, the former is not entirely representing the main cohort. The sub-cohort and those with the *AKR1C3* rs12529 CC and CG genotypes within the sub-cohort showed a negative correlation of BMI with age at PC diagnosis, although this was not seen in the main cohort except for a marginal negative trend shown among those carrying the *AKR1C3* rs12529 CC genotype in the main cohort. As age dependent BMI change is not directly relevant to the theme of this manuscript, it is considered towards the end of the discussion.

### Genotype frequency

The frequencies between the *AKR1C3* rs12529 genotypes are similar to that of the European Americans and African Americans recorded before [20, 28]. The frequency of the *AKR1C3* rs12529 G allele in our cohort was 0.463 which is marginally lower than the frequencies recorded in the Genome Aggregation Database (0.499); but similar to the Trans-Omics for Precision Medicine Database (0.476); and higher than the TWINSUK Database (0.385) and Avon Longitudinal Study of parents and Children Database from the University of Bristol (0.382) (https://www.ncbi.nlm.nih.gov/snp/rs12529). According to a study from Taiwan, the *AKR1C3* rs12529 G was the major allele (G = 0.988) in an Asian cohort [13].

### AKR1C3 activity

The median AKR1C3 activity measured in leukocytes was 0.41μM coumberol per million cells (25^th^ and 75^th^ percentiles at 0.21 and 0.73 respectively) and was approximately three times the level produced by the positive control LNCaP+ cell line (0.13μM coumberol per million cells, and 25^th^ and 75^th^ percentiles at 0.10 and 0.15 respectively).To our knowledge, this is the first recording of the AKR1C3 activity measurements made in leukocytes extracted from -80°C stored blood samples as well as in leukocytes from PC patients. When stratified by median age at PC diagnosis, men in ≥69y age group showed higher AKR1C3 activity compared to those at <69y. This is the first ever age based variation in leukocyte AKR1C3 activity recorded in men. However, when data were further stratified for the *AKR1C3* rs12529 genotype, none of the genotypes showed significant variation of AKR1C3 activity based on this age stratification. In this sub-cohort, AKR1C3 activity in leukocytes showed no variability with lifestyle factors (tobacco smoking, alcohol consumption and BMI). The *AKR1C3* promoter constructs of the HepG2 cells have previously shown that the promoter activity of this gene is associated with promoter SNP rs3763676 [15]. However, such information is not yet available for the *AKR1C3* rs12529 SNP.

Those with anatomic stage/prognostic group <IIB showed a higher AKR1C3 activity compared to those with ≥IIB group, when data were stratified by the *AKR1C3* rs12529 CC genotype. However, previous studies on AKR1C3 expression in PC tissue or PC cell lines have shown higher levels with PC progression and severity [29]. It is a possibility that the increase in AKR1C3 levels with disease severity are either confined to PC tissue/cell lines or such hierarchical increases are delegated to PC tissue at the expense of AKR1C3 activity elsewhere such as is seen in the leukocytes. If the latter is the case, the reduced levels in AKR1C3 activity in leukocytes among men with the *AKR1C3* rs12529 CC genotype may be a reflection of increased levels of AKR1C3 in PC tissue. However, this has to be verified in future studies. Meanwhile, hierarchy in AKR1C3 production between various tissue types has been recorded before [30]. It has been shown that castration resistance is associated with increased expression of genes including that of AKR1C3 [17]. In the current analysis, AKR1C3 activity in leukocytes of men stratified between those who have received ADT and those managed without ADT showed no variation. It could be either due to AKR1C3 over-expression with castration by ADTs being limited to PC cells or due to such increases being limited to castration resistant PC patients. However, as these men were recruited from Urology clinics, there was only 1.8% and 0.8% of patients from the main and sub-cohorts recording castrate -resistant PC. Due to the small sample size used for the leukocyte AKR1C3 activity measurement, the ADT group included both short- and long-term treatment groups and thereby, diluting the possible long-term ADT impacts on AKR1C3 activity. AKR1C3 is also known to be produced in subcutaneous fat deposits, especially in obese women and those with polycystic ovarian syndrome, and considered to be a factor towards intra-adipose testosterone and DHT [31]. However, as men in this sub-cohort showed a decrease in BMI with increasing age, which could be associated with a decrease in fat deposits, AKR1C3 produced in subcutaneous fat compartments would have diminished with age. It is interesting to know in the future whether this decline in subcutaneous fat-based AKR1C3 production is compensated by the production in leukocyte based AKR1C3, with increasing age. An interesting observation made in this analysis is that the leukocyte AKR1C3 activity was significantly associated with age in the sub-cohort. Genetic stratification shows that this trend in age at diagnosis and leukocyte AKR1C3 activity correlation is seen only in men with the *AKR1C3* rs12529 GG genotype (correlation coefficient 0.35, P = 0.09). It is a possibility that with increasing age, men with the *AKR1C3* rs12529 GG genotype have a potential to produce higher AKR1C3 activity compared to those with other genotypes, and subsequently support a higher proportion of extra-testicular androgen production that increases the androgen pool.

### Clinical parameters and AKR1C3 rs12529 genotypes

In the main cohort ever-smokers showed a marginally significant increase in age at diagnosis compared to never-smokers. When this feature was stratified by genotypes, it is the *AKR1C3* rs12529 GG genotype which shows a prominent increase in age at diagnosis among ever-smokers compared to never- smokers. In the main cohort, PSA at diagnosis was not significantly different between ever- and never-smokers. However, when stratified by genotypes it was only the *AKR1C3* rs12529 CG genotype that showed a significantly higher PSA at diagnosis among ever-smokers compared to never-smokers. Involvement of AKR1C3 in metabolizing polycyclic aromatic hydrocarbons (PAH) leading to the formation of pro-reactive oxygen species such as catechols and quinones have been documented [32–34]. Meanwhile, Lan et al 2004 have recorded that the *AKR1C3* rs12529 GG genotype was more susceptible to lung cancer risk in those exposed to smoke derived from coal burning [35].Tobacco smoke is known for its PAH content and the tobacco smoke exposure-related PAH kinetics have been studied in humans previously [36]. Impacts of a 3 mg/kg dose of tobacco smoke constituent bezo-a pyrene given on five occasions within a 26 day period to male Tilapia fish is reported by Colli-Dula *et al* 2018 [37]. The authors report a gene ontology analysis of PAH effects and report changes including that of AR to PSA signalling pathway that was shown to get up-regulated in the liver samples (1.35 fold) and decrease in testis samples (1.05 fold). They also show a 1.8 fold increase in glutathione peroxidase transcripts in liver tissue by BaP treatment without any changes in the testes. Rybicki *et al* 2008 [38] have evaluated the proportion of PAH-DNA adduct levels in both tumour and non-tumour cells from surgical prostate tissue. These authors record that after one year follow up from surgery, there is a transient association in the number of men with biochemical recurrence (BCR) with that of PAH-DNA adduct levels in both tumour and non-tumour tissue. These authors also report that a higher level of adducts in non-tumour tissue compared to tumour tissue led to stronger association with BCR, reflecting an innate ability of the non-tumour tissue to activate carcinogens. It is possible that PC patients carrying the AKR1C3 rs12529 G alleles have a higher impact of tobacco smoking affecting their PSA based PC diagnosis. We have previously recorded delayed diagnosis of high-risk PC in ever-smokers from NZ carrying the *AKR1C3* rs12529 G allele when compared to similar cohorts from the US [20]. Elimination of those with the *AKR1C3* rs12529 G allele for screen detected PC due to their lower PSA levels is a possibility with a subsequent delayed diagnosis with high-risk PC. Those carrying the *AKR1C3* rs12529 CG genotype getting diagnosed at a higher PSA level especially among ever-smokers as reported here could also be due to these men not getting captured earlier with low risk PC at a lower PSA level. For those carrying the *AKR1C3* rs12529 G allele and are ever-smokers, their AKR1C3 activity may not be able to catalyse the production of extra-testicular androgens at the same rate as their never-smoker counterparts and those with the CC genotype and are ever-smokers.

In both the sub- and main cohort, the age at diagnosis significantly correlated with the PSA at diagnosis. However, it was shown to be relevant only to those with the *AKR1C3* rs12529 CG and GG genotypes and not the CC genotype. It is a possibility that for at least the *AKR1C3* rs12529 GG genotype carriers, this may be related to their potential to produce higher levels of AKR1C3 activity with age. This can be also interpreted as those with the *AKR1C3* rs12529 G alleles could have significant PCs that go undetected at lower ages due to lower PSA levels. It has been shown that physiological levels of DHT (10nm) treatment in LNCaP PC cell line, causes a 70% reduction in AKR1C3 activity [39] that can be interpreted as lower levels promoting AKR1C3 activity. Therefore, a hypothesis that can be derived from these results is that for those with the *AKR1C3* rs12529 CG and GG genotypes, general reduction in androgen around 65-75y [40, 41] promote AKR1C3 activity based extra-testicular androgen production while for the CC genotype carriers, this cannot take place in this age range. If this correlation between age and PSA at diagnosis can be proven in larger cohorts elsewhere, this may prove to be among the solutions to improve age based PSA thresholds for PC screening. If this fact cannot be proven in larger cohorts elsewhere, it could be a NZ specific factor/s. We have previously recorded that our NZ PC cohort were diagnosed at higher age and PSA levels compared to that of African and Caucasian PC cohorts from US [20]. NZ has very specific conditions that might especially impact ever-smokers, including the relatively lower levels of available dietary selenium (Se) when compared to most other regions in the world. Serum Se levels are relatively lower in both PC patients and healthy men in NZ compared to levels in certain other parts of the world [42, 43]. Additionally, both low serum Se and tobacco smoking have come up as risk factors for PC incidence in our studies with NZ men [43] while the latter came up as a risk factor for its high-risk PC forms in our NZ cohort [5]. A systematic review and meta-analysis show that tobacco smoking was associated with PC incidence in the era prior to PSA based screening for PC but not since then [44], meaning that NZ may carry a unique PC risk status with regards to the environmental factors such as tobacco smoking. Our studies with NZ cohorts have also shown that current smokers among men with no diagnosis of PC have lower levels of serum Se compared to never- smokers [45] and that the seleno-enzyme glutathione peroxidase level increases almost two fold when 200μg Se is supplemented for six months particularly in ever-smokers compared to never-smokers in NZ [46].

In both the sub- and main cohorts, PSA at diagnosis and the Gleason sum correlated significantly including when stratified by the *AKR1C3* rs12529 CC and CG genotypes. This was also the same with the GG genotype group in the main cohort. A similar PSA at diagnosis and Gleason sum correlation has been reported by Yarney et al 2013 for an African cohort with a mean age at presentation at 65.4y [47]. Thompson *et al* 2006 have reported PSA level as a predictive factor for high-grade disease (Gleason score ≥ 7) [48]. A study with a cohort of African men with a mean age at diagnosis >70y, Gleason sum has been assessed for linear correlation with PSA at diagnosis [49]. Although these authors record no linear correlation, they have not assessed the non-linear correlation between the Gleason sum and PSA at PC presentation in this group. Correlation coefficients between PSA at diagnosis and Gleason sum were stronger in the current sub-cohort, indicating that this correlation increases with increasing severity of the disease.

### BMI and age

BMI increases from young age to middle age due to increasing fat mass and subsequent decrease from middle to old age due to decrease in lean mass are well known [50–52]. Therefore, the decrease in BMI with increasing age in the older sub-cohort is as expected. Additionally, the discrepancy of age based BMI decline between the sub- and the main cohorts can be attributed to variation in recruitment criteria between initial and late stages of the study. The time lag between diagnosis to recruitment being similar in the sub- and main cohorts, we cannot assume that the age at diagnosis dependent BMI decline in the sub-cohort to be associated with this factor. Instead, the age at diagnosis dependent BMI decline could also be associated with the clinical features and PC management options between the sub- and main cohorts. van Londen *et al* 2008 [53] have monitored body fat and lean mass in a group of PC patients either receiving ADT or not and a group of healthy men, over a period of 24 months. They have shown that % body fat from total body mass increases by approximately 2% after acute ADT (ADT initiation <six months prior to enrolment) and approximately 1% in the chronic ADT (treated with ADT for ≥six months at enrolment) after 24 months. They also report a reduction in the proportion of lean body mass to total body mass by 2% in the acute ADT group and 1% in the chronic ADT group after 24 months of enrolment. In their study the impact on PC patients not receiving ADT were less than 0.5% gain in body fat from total body mass as well and less than 0.5% loss in lean body mass proportion from total body mass, 24 months from enrolment. However, there was no deficit between weight gain by fat mass and decline in lean mass due to ADT. However, BMI of these different groups at enrolment in van Londen *et al* [53] study has remained comparable. A systematic review also shows an increase in percentage body fat and decrease in percentage lean mass in PC patients treated with ADT [54]. Age-related bone loss is reported in PC patients not receiving ADT [55]. Bone mineral density decline in PC patients receiving ADT is also reported [56, 57]. However, the majority of studies indicate that BMD is negatively associated with BMI, while some showing a ‘U’ shaped relationship between BMI and bone fracture risk [58, 59]. Therefore, it is not possible to relate BMI decline with increasing age in the current sub-cohort as due to ADT.

### Conclusions

Although PSA was considered as the gold standard for screening for PC, it has reached a controversial status since 2008 [60, 61]. This is due to both over-diagnosis and over-treatment of men as well as under-diagnosis of others when screen diagnosed with PC. Our assessment shows that age based PSA increase in men carrying PC is limited to those with the *AKR1C3* rs12529 CG and GG genotypes. This means for those men carrying the *AKR1C3* rs12529 CC genotype (which is 32% of our cohort), an age based PSA increase is not a valid concept. Age dependant PSA correlation was stronger in the sub-cohort that recorded more severe disease. When this sub-cohort was stratified, it was only the AKR1C3 rs12529 CG (correlation coefficient = 0.46, P<0.01) and the GG (0.39, P = 0.06) genotypes that showed correlation between age and PSA at diagnosis. In this sub-cohort and the AKR1C3 rs12529 CG genotype of this sub-cohort, as well as the GG genotype of the main cohort, age dependant correlation of Gleason sum was also significant. It is a possibility that with increasing age, men carrying the *AKR1C3* rs12529 G allele has an increasing potential to produce more AKR1C3 activity, thereby adding a higher proportion of adrenal derived extra-testicular androgen to the androgen pool. Such increases could be the reason behind age dependant PSA increases seen in the *AKR1C3* rs12529 G allele carriers. The *AKR1C3* rs12529 G allele is the major allele in Asian, Maori and Pacific men in our cohort [62]. Therefore, it will be of major importance to come up with a new set of age-based PSA cut-off thresholds for PC screening especially for these men as well as all NZ men carrying the *AKR1C3* rs12529 G allele. Among the draw backs in our study are the small sample size used for the leukocyte AKR1C3 measurement; pooling all types of ADT based management options under one category in our assessment and non-availability of TNM staging for 36% and 23% respectively for the main and sub-cohorts. It is possible with a significantly larger sample size, with better patient stratification, a better assessment between genotype based leukocyte AKR1C3 activities can be confirmed and compared against biological, lifestyle and clinical features and pave way for a more stringent genetically stratified PSA-based PC screening.

## Supporting information

S1 Table Summary of details related to batch genotyping for the *AKR1C3* rs12529 SNP. (Details provided as a requirement of the Standard Strengthening the Reporting of Genetic Association Studies (STREGA)–An Extension of the STROBE Statement.).
(DOCX)

S2 Table Supplementary microsoft office excel data sheet. (XLSX)

## Abbreviations

AJCC: American Joint Committee on Cancer
AKR1C3: Aldo-keto reductase 1C3
EDTA: Ethylenediaminetetraacetic acid
NZ: New Zealand
PBS: Phosphate buffered saline
PC: Prostate cancer
PCR: Polymerase chain reaction
PSA: Prostate-specific antigen
SNP: Single nucleotide polymorphism
TNM: Tumor–Node–Metastasis
