# Supplementary material for: Interaction between leukocyte aldo-keto reductase 1C3 activity, genotypes, biological, lifestyle and clinical features in a prostate cancer cohort from New Zealand
Source: PLoS One. 2019 May 24;14(5):e0217373. doi: 10.1371/journal.pone.0217373 (PMC6534310; doi:10.1371/journal.pone.0217373)
Supplement: S1 Table — (Details provided as a requirement of the Standard Strengthening the Reporting of Genetic Association Studies (STREGA)–An Extension of the STROBE Statement.). (DOCX) [file pone.0217373.s001.docx]

**Supplementary Table S2 -Summary of details related to batch genotyping for the *AKR1C3* rs12529 SNP**

(Details provided as a requirement of the Standard Strengthening the Reporting of Genetic Association Studies (STREGA)–An Extension of the STROBE Statement.)

| **1a. DNA extraction, quantitation and purity checking** | |
| --- | --- |
| Total genomic DNA extraction | *NZ cohort*-  QIAamp DNA Blood Mini Kit (Catalogue # 51106 from Qiagen, Hilden, Germany) according to the manufacturer’s instructions using a fully automated procedure on the QIAcube -230V (Catalogue # 9001293 from Qiagen, Hilden, Germany). |
| DNA quantitation and purity checking and dilution | NanoDrop 1000 v 3.8. ( Thermo Scientific, Wilmington, USA) |
| DNA storage and dilution for genotyping | DNA samples were stored at -20^0^C. All DNA samples were normalised to 10ng µl^-1^. |

| **1b. Genotyping methods** | | |
| --- | --- | --- |
| **Method** | Sequenom’s genotyping iPLEX Assay (Sequenom)* | TaqMan SNP Genotyping (Applied Biosystems)** |
| Primer design | MassArray Assay Design Software v 3.0 was used to design a multiplex SNP genotyping assay that included the AKR1C3 rs12529 | Predesigned by assay on demand (C__8723970_1_from the Applied Biosystems |
| Genotyping assay | According to manufacturer’s specifications (Sequenom, San Diego, CA, USA) | Reactions prepared based on Ferguson et al ^1^ |
| Detection method | Matrix-assisted laser  desorption/ionization-time of flight  (MALDI TOF) mass spectrometry.  (Spotting and firing of samples were carried out at AgResearch Ltd, Invermay Agricultural Centre, Puddle Alley, Mosgiel, New Zealand.) | Sequence detection system SDS v 2^.^4 software.  These were carried out at the Auckland Cancer Society Research Centre, University of Auckland, |
| Genotype calling software | iPLEX MassARRAY  Typer v.4^.^0 software |  |

| **1c. Details of batch genotyping** | | | | |
| --- | --- | --- | --- | --- |
| Batch | Operator/method | DNA Storage time in -20^0^C before genotyping | Total number assayed | Total produced results  (% call rate) |
| *All Urology patients (with prostate cancer and with benign urology disease) genotyped in NZ* | | | | |
| NZ 1 | Katja Lange- Sequenom | 2y 2m | 178 | 176 (98^.^9) |
| NZ 2 | Alice Wang- TaqMan | 3y 2m | 313 | 305 (97^.^4) |
| NZ 3 | Alice Wang - TaqMan | 1y 3m | 96 | 96 (100) |
| NZ 4 | Alice Wang - TaqMan | <1y | 45 | 43 (95^.^6) |
| NZ 5 | Alice Wang- TaqMan | <1y | 19 | 19 (100) |

| **1d. Batch genotype and allele numbers** | | | | | | |
| --- | --- | --- | --- | --- | --- | --- |
| Batch | Observed genotype numbers | | | | Observed allele numbers | |
|  | CC | CG | GG | Total | C | G |
| NZ 1 | 53 | 82 | 41 | 176 | 188 | 164 |
| NZ 2 | 107 | 136 | 62 | 305 | 350 | 260 |
| NZ 3 | 32 | 40 | 24 | 96 | 104 | 88 |
| NZ 4 | 12 | 20 | 11 | 43 | 44 | 42 |
| NZ 5 | 6 | 9 | 4 | 19 | 21 | 17 |

| **1e. Observed and expected allele frequencies from each batch** | | | | | |
| --- | --- | --- | --- | --- | --- |
| Batch | Observed allele frequencies | | Expected genotype frequencies | | |
|  | C | G | CC | CG | GG |
| NZ 1 | 0^.^53 | 0^.^47 | 0^.^29 | 0^.^50 | 0^.^22 |
| NZ 2 | 0^.^57 | 0^.^43 | 0^.^33 | 0^.^49 | 0^.^18 |
| NZ 3 | 0^.^54 | 0^.^46 | 0^.^29 | 0^.^50 | 0^.^21 |
| NZ 4 | 0^.^51 | 0^.^49 | 0^.^26 | 0^.^50 | 0^.^24 |
| NZ 5 | 0^.^55 | 0^.^45 | 0^.^31 | 0^.^49 | 0^.^20 |

| **1f. Expected genotype numbers and Hardy-Weinberg equilibrium*** statistics for each batch. (Significance set at p<0.05)** | | | | |
| --- | --- | --- | --- | --- |
| Batch | Expected genotype numbers | | | p-value |
|  | C/C | CG | GG |  |
| NZ 1 | 50^.^20 | 87^.^59 | 38^.^20 | 0^.^40 |
| NZ 2 | 100^.^41 | 149^.^18 | 55^.^41 | 0^.^12 |
| NZ 3 | 28^.^17 | 47^.^67 | 20^.^17 | 0^.^12 |
| NZ 4 | 11^.^26 | 21^.^49 | 10^.^26 | 0^.^65 |
| NZ 5 | 5^.^80 | 9^.^39 | 3^.^80 | 0^.^85 |

| **1g. Availability of the *AKR1C3 rs12529* genotype data matched to clinical data** | | | |
| --- | --- | --- | --- |
| *AKR1C3 rs12529* genotypes | CC | CG | GG |
| NZ | 119 | 166 | 91 |

DNA extraction from NZ cohort was carried out using the QIAamp DNA Blood Mini Kit (Catalogue # 51106 from Qiagen, Hilden, Germany). A fully automated procedure on the QIAcube -230V (Catalogue # 9001293 from Qiagen, Hilden, Germany) was followed according to the manufacturer’s recommendations. Extracted DNA was checked for the quality and quantity using the NanoDrop 1000 v 3.8. (Thermo Scientific, Wilmington, USA), and stored at -20^0^C. All DNA samples were normalised to 10ng µl^-1^.

*The first batch (NZ1) of genotyping was carried out using Sequenom MassArray and iPlex system^2, 3^ according to manufacturer’s specifications (Sequenom, San Diego, CA, USA). The assay of *AKR1C3* rs12529 SNP was analysed as part of a multiplexed SNP genotype assay to evaluate association of androgen pathway related SNPs for the risk of PC. The MassArray Assay Design Software v 3^.^0 was used to design a multiplex SNP genotyping assay. The subsequent procedures including assay optimisation, PCR reactions, cleaning of the amplified product, allele discrimination reactions, spotting onto SpectroCHIP microarray, and MALDI-TOF mass spectrometry are described elsewhere.^1, 4^ A total of 8 no-template controls (NTC), 10 HapMap controls (Centre d’Etude du polymorphism Human (CEPH) samples from Utah residents with ancestry from northern and western Europe), test samples, and 8 duplicate samples were assayed in one plate. Spotting and firing of samples were carried out at AgResearch Ltd, Invermay Agricultural Centre, Puddle Alley, Mosgiel, New Zealand. Data analysis was carried out using the iPLEX MassARRAY Typer v.4^.^0 software. Calls on HapMap control samples in this multiplex assay were 99^.^4% identical with data given in HapMap Genome Browser release #28 (<http://hapmap.ncbi.nlm.nih.gov>).

**The rest of NZ patients were genotyped using the TaqMan® SNP Genotyping Assay using allele-specific, dual-labelled hybridization probes [predesigned by assay on demand (C__8723970_1_from the Applied Biosystems]. The reaction preparation is described in Ferguson *et al*.^1^ At assay optimisation a total of 4 NTC and 20 HapMap CEPH controls were assayed. Thereafter, each batch was assayed with 7-8 NTC, 6-8 HapMap CEPH controls, and 3-4 duplicate samples except in the analysis of the last batch where only two duplicate samples were used. The assay was run on the Applied Biosystems 7900HT Fast Real-Time PCR System at the Faculty of Medical and Health Sciences, the University of Auckland. The PCR conditions for the assay were: 10 min 95 °C enzyme activation followed by 40 cycles at 92 °C for 15 s and 60 °C for 1 min (annealing/extension). The allelic discrimination results were determined after amplification by performing an endpoint read using sequence detection system SDS v 2^.^4 software. Calls on HapMap control samples were 100% identical with data given in HapMap Genome Browser release #28 (<http://hapmap.ncbi.nlm.nih.gov>). Genotyping call rate for test samples varied between 95^.^6-100%. Genotype data collected with the above procedures from our previous studies^5^ as well as updated genotype data collections were matched to 379 patients from the current study. (Supplementary Data file).

***Genotype data for the all batches were assessed for the Hardy-Weinberg Equilibrium using a two allele system applying the binomial theorem.^6^

1. Ferguson LR, Han DY, Fraser AG, et al. Genetic factors in chronic inflammation: single nucleotide polymorphisms in the STAT-JAK pathway, susceptibility to DNA damage and Crohn's disease in a New Zealand population. *Mutat Res* 2010; **690**(1-2): 108-15.

2. Jurinke C, van den Boom D, Cantor CR, Koster H. The use of MassARRAY technology for high throughput genotyping. *Adv Biochem Eng Biotechnol* 2002; **77**: 57-74.

3. Storm N, Darnhofer-Patel B, van den Boom D, Rodi CP. MALDI-TOF mass spectrometry-based SNP genotyping. *Methods Mol Biol* 2003; **212**: 241-62.

4. Morgan AR, Fraser AG, Ferguson LR. Metallothionein genes: no association with Crohn's disease in a New Zealand population. J Negat Results Biomed 2012; 11: 8. doi: 10.1186/1477-5751-11-8.5. Karunasinghe N, Lange K, Han D, et al. Androgen pathway related gene variants and prostate cancer association in Auckland men *Curr Pharmacogenomics Person Med* 2013; **11** (1): 22–30.

6. Edwards AW. G. H. Hardy (1908) and Hardy-Weinberg equilibrium. *Genetics* 2008; **179**(3): 1143–50.
